# Fifteen-year recall period on zirconia-based single crowns and fixed dental prostheses. A prospective observational study

**DOI:** 10.1038/s41405-024-00214-7

**Published:** 2024-06-20

**Authors:** Shahnawaz Khijmatgar, Margherita Tumedei, Guilia Tartaglia, Michele Crescentini, Gaetano Isola, Ernesto Sidoti, Chiarella Sforza, Massimo Del Fabbro, Gianluca Martino Tartaglia

**Affiliations:** 1 Fondazione IRCCS Ca’ Granda Ospedale Maggiore Policlinico, Milan, Italy; 2 School of Medicine, University of Madrid, Madrid, Spain; 3 Department of General Surgery and Surgical-Medical Specialties, School of Dentistry, University of Catania, Via S. Sofia 78, 95124 Catania, Italy; 4 SST Clinic Private Practice Segrate, Milano, Italy; 5 Department of Biomedical Sciences for Health, University of Milan, Via Mangiagalli 31, 20133 Milan, Italy

**Keywords:** Fixed prosthodontics, Bonded restorations

## Abstract

**Aim:**

The aim of this study was to evaluate the long-term clinical outcomes of zirconia-based prostheses used for tooth-supported or implant-supported single crowns and fixed dental prostheses (FPD).

**Methods:**

The authors conducted a prospective analysis of 562 zirconia core restorations supported by endodontically treated teeth or titanium implant in 276 patients in a general dental private practice, with a follow-up period of 15 years. The study was stopped after patients achieved 15 yrs of follow-up. The study analyzed the failure and complication rates of single and multiple crowns, based on Kaplan Meier analysis.

**Results:**

During follow-up period, there were 26 complications and 156 failures. The crown level analysis revealed a cumulative failure rate of 28.33% and complication rate of 8.47% for zirconia crowns after 15 years. The complication rate was found to be higher for titanium implant-supported than for natural teeth-supported crowns. The different types of crown-based failure include: veener fracture 5.01% (*N* = 29), metal zirconia led to 14.85% (*N* = 86) loss of retention, and 1.73% (*N* = 10) loss of crown due to extraction.

**Conclusion:**

Based on these findings, zirconia core restorations appear to be a reliable long-term solution for crowns and fixed dental prostheses.

**Clinical relevance:**

The study suggests that zirconia restorations can be successfully used for long-term prostheses on natural teeth or implants supported. The study results provide clinicians valuable information when selecting prosthetic restorations material.

## Introduction

The zirconia-based restorations symbolise a new standard for dental restorations and fixed dental prosthesis (FDP) due to their high loading resistance and aesthetics compared to conventional metal-ceramic prostheses. The main physical characteristics are interconnected to an increased flexural resistance and fracture strength and hardness. This produce a high tolerance to the cyclical loading and low surface wearing with a high biocompatibility [1]. Moreover, the aesthetic integration could be improved with the surface veneering of the zirconia cores, especially in the frontal region of the maxillary and mandibular arches [1–3]. The zirconia-based materials are characterized by the ability to avoid the crack propagation generated by the surface loading stresses due to the change of the poly-crystalline structure [4, 5]. This ensures optimal stability and longevity of the zirconia crowns, minimizing the risk of complications such as microleakage, inflammation, and potential damage to the surrounding tissues. Additionally, a precise passive fit promotes better occlusal harmony and patient comfort, contributing to the overall success of the rehabilitation [6]. Our previous study reported a 7-year cumulative survival rate of 94.7% for zirconia dental rehabilitations on natural teeth and implants [7]. The most common challenges of zirconia crowns failure are chipping, cracking, delamination of the veneering-to-core level, alteration of the surface colour [8, 9].

Konstantinidis et al. [10] study aimed to compare the 3-year performance of implant-retained and tooth-retained zirconia-based fixed dental prostheses (FDPs) with at least 4 units. The null hypothesis was that complication rates in both groups would be equally distributed. The study included 20 patients with tooth-retained and 7 with implant-retained FDPs. Chipping rates in the implant group (71%) were significantly higher than in the tooth group (15%). Unit-related chipping rates were also higher in the implant group. Despite satisfactory overall survival rates, the study suggests that long-span implant-retained FDPs with zirconia frameworks may be discouraged due to high veneering ceramic chipping rates [10]. Another retrospective study by Tanner et al. [11] aimed to assess the survival and complications of zirconia crowns and FDPs. The overall survival rate for zirconia restorations was 95%, with single crowns at 94.2% and FDPs at 95.7%. Complication rates were 26% for FDPs and 5.8% for crowns. The most common issues were veneering ceramic fractures (12%) and bleeding on probing (38.1% for restored teeth) [11].

A prospective observational study would be an appropriate study design for this type of research as it involves following a group of patients who receive zirconia-based dental crowns over a period and monitoring their outcomes. By collecting data on the success rates of the crowns and any complications that may arise, researchers can evaluate the performance of zirconia-based dental crowns as fixed dental prostheses. Literature has reports related to longitudinal data especially for mid- and short-term periods that may help in understanding the immediate or early causes of failures and modify the procedures [7]. Instead, long-term results would be beneficial for other reasons. First, it would provide valuable information to dental professionals on the long-term durability and success rates of zirconia-based dental crowns. This information could valuably support clinical decision-making and secondly, help dentists choose the most appropriate materials for their patients’ restorations, and enhance patients’ satisfaction.

Moreover, the study could help identify any potential complications or adverse events associated with zirconia-based dental crowns. This information could help improvements in the design and manufacturing of these restorations. Generally, a 15-year prospective clinical study on zirconia-based single crowns and FDPs would provide valuable information to improve patient outcomes, and ultimately advance the field of dental prosthetics. Therefore, the aim of the present prospective study was to evaluate the cumulative survival/failure rates and the complication rate of zirconia dental restorations after 15-years follow up.

## Materials and methods

The study was conducted in compliance with the Helsinki Declaration for Medical Research. Prior to the investigation’s second phase, patients were notified and gave their written consent to participate. All participants had previously given their written informed consent for clinical procedures in accordance with the existing guidelines for good clinical practice and the current Italian Law. No prior ethical approval was required as this was an observational study of standard clinical treatments with no experimental intervention. Patients consented and who had visited the dental clinic between 2006 and 2008 were included in the study.

The inclusion criteria have been described in a previous report [7]. Subjects received one or more zirconia-based crowns supported by implants or endodontically treated teeth. Patients were excluded if they had non-surgical endodontic re-treatment on teeth that had radiographic evidence of broken instruments, endodontic canal overfilling and incorrect working length, mechanical perforations in the absence of peri-radicular diseases, and periodontal disease. Patients with compromised medical condition (i.e. cancer, uncontrolled diabetes, immunodepression) were also excluded. Subjects who signed the informed consent form for the clinical procedures and research recruitment that reported being in good medical health were included.

The included subjects were evaluated by an independent clinician that had not been involved in the original prosthetic procedures. “The survival rate was defined as surviving fixed dental prosthesis (FDPs) minus altered FDPs based on two (grades 2 and 3) of the three grades scale of chipping fractures”v[12]. Surface chipping is “graded 1” if the fractured surface is not extended into a functional area and polishing is possible. Recontouring will result in an acceptable alteration of the anatomic form from the original anatomy. “Grade 2” indicates moderate surface chipping and “Grade 3” indicates severe veneer ceramic chipping with exposure to the zirconia core [13]. “Failure” is defined as the complete and irreversible loss of function, integrity, or structural stability of the zirconia crown. “Complication” in zirconia dental crowns prosthesis refers to issues or challenges that may arise during or after the placement of the crown but do not result in the complete loss of function or structural integrity [12].

### Zirconia-based crowns

All evaluated teeth had a tooth restorability index of 2 or less, indicating insufficient residual coronal dentine for restoration according to operator judgment. Composite material (LuxaCore, DMG, Hamburg, Germany) was used to manufacture dental cores when the occlusal space measured less than 2 mm in centric occlusion. Residual abutments needed to be at least 4 mm high from the buccal and lingual gingival margins to the occlusal surface. Multiple three to six-unit restorations had a total gap equal to or less than the crown-root surface area of the abutment teeth being restored, with a minimum of 3 mm of occluso-gingival height. A specially made tray was used to take impressions of teeth and implants (using the pick-up technique) using polyether (Impregum/Permadyne, 3 M ESPE AG, Seefeld, Germany). ZOE (temporary zinc oxide-eugenol) cement (Temp Bond, Kerr Italia, Scafati, Salerno, Italia) was used to cement customised provisional resin crowns (Takilon BB, Salmoiraghi srl, Melegnano, Lodi, Italia). A laser scanner, was used to scan a plaster model (Esthetic-base gold, Dentona AG, Dormund, Germany) and used to create an anatomical contour wax-up. Zirconia cores were designed with respect to ceramic support and fabricated from pre-sintered state using Computer-Aided Manufacturing (CAM) and sintered according to manufacturer instructions. Feldspathic porcelain (CZR Noritake Kizai Co. Ldt., Nagoya, Japan) was fused onto the cores by a master ceramist [14–16]. Proximal and occlusal contacts were adjusted as needed. Abutment teeth or implants were cleaned before cementation using glass-ionomer cement (Ketac, 3 M ESPE AG, Seefeld, Germany) for final cementation. Functional analysis of masticatory muscles was conducted before and after cementation of final prostheses following a standardized protocol. This analysis was required for patients with more than 4 unit crown/bridge restorations, and all included patients demonstrated good neuromuscular equilibrium. These methods align with those outlined in earlier follow-up studies (7,16).

### Statistical analysis

Sample Size calculation: To estimate the required sample size for the study power analysis calculation was performed using G power software [17]. The sample size calculation formula for a two-sample t-test used was [17]:

$${{{{{\rm{n}}}}}}={2}* {({{{{{\rm{Z}}}}}}\_{{{{{\rm{alpha}}}}}}/2+{{{{{\rm{Z}}}}}}\_{{{{{\rm{beta}}}}}})}^{2}* {({{{{{\rm{S}}}}}}/{{{{{\rm{ES}}}}}})}^{2}$$

Where:

${{{{{\rm{{Z}}}}}\_{alpha}}}/2$ is the critical value of the standard normal distribution at alpha/2 level of significance (two-tailed)

${{{{{\rm{{Z}}}}}\_{beta}}}$ is the critical value of the standard normal distribution at the desired power level

S is the pooled standard deviation of the two groups

ES is the effect size (difference in means divided by the standard deviation)

Assuming a two-sample t-test and a 1:1 ratio of treatment groups, with an effect size of 0.8, alpha level of 0.05, and power of 0.8, the critical values of the standard normal distribution are:

$${{{{{\rm{{Z}}}}}\_{alpha}}}/2=1.96$$

$${{{{{\rm{{Z}}}}}\_{beta}}}=0.84$$

The pooled standard deviation (S) is not known and needs to be estimated based on previous research. Assuming a conservative estimate of *S* = 10, and substituting these values into the formula, will get:

$${{{{{\rm{n}}}}}}=2* {(1.96+0.84)}^{2}* {(10/0.8)}^{2}=34.27$$

Therefore, the estimated sample size for this study is 34 participants per group (crown on natural teeth vs dental implants), or a total sample size of 68 participants. For the investigated group of zirconia crowns, Kaplan-Meier estimates of overall survival time and complication-free rate time were determined using two types of comparison, crown and patient levels. The approach calculated the percentage of crowns that lasted for a set period following final cementation, considering the effect of lost to follow-up (or censored) crowns. Patients were censored in survival analysis if they had not experienced the endpoint of interest at the time of the study.

The hazard ratios (HR) and 95% confidence intervals (CI) were determined. Graphical inspection and statistical testing were used to evaluate the model assumption of proportional risks. Given the collinearity between gender, type of support, and number of crowns (all events fall into only one of these categories), no multivariable model was built.

To facilitate comparison with earlier studies, the cumulative survival rate (CSR) was estimated using the life table technique. For all the above studies, statistical significance was established at 5% (p value 0.05).

The term “time-to-event analysis” refers to a set of methodologies for examining the amount of time until a well-defined end point of interest occurs. The final exit time was reported for both failures and complication at crown and patient levels data.

A cluster analysis using factor loading was conducted. A factor loading plot in cluster analysis is a visual representation that shows how strongly each variable (factor) contributes to the formation of different clusters. It helps identify which factors are most influential in determining cluster membership. The plot enables in understanding which variables are most relevant in distinguishing or grouping patients, conditions, or other dental-related entities into distinct clusters. It provides insights into the key factors driving the clustering patterns. When examining a factor loading plot, variables with high positive or negative loadings have a strong association with specific clusters. These variables contribute the most to the formation of those clusters. On the other hand, variables with loadings close to zero have little influence on the cluster formation. Interpreting the plot involves looking for patterns, such as variables clustering together or being more closely associated with certain clusters. STATA Version 17.0 USA statistical software was used for survival analysis.

## Results

The study analyzed a total of *N* = 562 dental crowns (Table 1), which were classified into two categories: zirconia crowns on natural teeth (*N* = 378) and implant-based crowns (*N* = 184) (Table 2). The data was prospectively collected and had a mean age of 66 ± 01 years (95% CI 64.4, 67.6), with *N* = 205 females and *N* = 173 males in the natural teeth group, and *N* = 114 females and *N* = 70 males in the dental implants group. The maxillary arch included *N* = 306 and mandible *N* = 256 zirconia crowns (Supplementary Table S1).

**Table 1:** Descriptive Statistics for failures based on crown-based data.

	Descriptive Statistics for Failure			
	*N*	Mean	Min-max	median
Number of subjects	276	−	−	−
Number of Crowns	562	−	−	−
Exit time	−	151	4.3–216	168
Time at Risk	84618	151	4.3–216	168
Failures	156	−	−	−

**Table 2:** Zirconia Crown Based descriptive analysis for both group and arch for failure as outcome.

Group	Arch	Number of Crowns	Exit time final (Months)		Time at risk	Failures	Complications
		Total (N)	Mean (min-max)	Median	Total Months	*N*	*N*
Natural teeth Women	Maxillary Anterior	29	166.3 (49.4–188.5)	184.6	4820	5	2
	Maxillary Posterior	80	151.17 (24.8–193.1)	168.35	12073	25	2
	Mandibular Anterior	8	152.45 (49.4–176.9)	167.6	1219.6	1	0
	Mandibular posterior	87	156.0 (26.0–216)	174.7	13571	17	4
Implant Women	Maxillary Anterior	12	136.19 (75.4–175)	162.6	1634.3	4	1
	Maxillary Posterior	37	140.2 (40.7–184.6)	157.6	5188.5	13	7
	Mandibular Anterior	10	165.85 (149.4–176.6)	165.6	1658.6	0	1
	Mandibular posterior	54	145.7 (23.7–190.3)	165.5	7872	16	7
Natural Teeth Men	Maxillary Anterior	25	157.4 (32.1–184.2)	167.4	3936.0	5	1
	Maxillary Posterior	82	146.5 (11.1-191.8)	169.2	12018.8	30	0
	Mandibular Anterior	2	140.4 (140.4)	140.4	140.4	1	0
	Mandibular posterior	65	154.18 (40.2–191)	172.1	10022.3	17	0
Implant men	Maxillary Anterior	6	188.45 (168.9–202.4)	187.2	1130.7	0	0
	Maxillary Posterior	35	149.8 (4.3–186)	169.2	5243.1	10	0
	Mandibular Anterior	2	172.8 (172.8)	172.8	172.8	0	0
	Mandibular posterior	28	132.5 (26.6–189.6)	132.5	3711.4	11	1

**Fig. 1:**
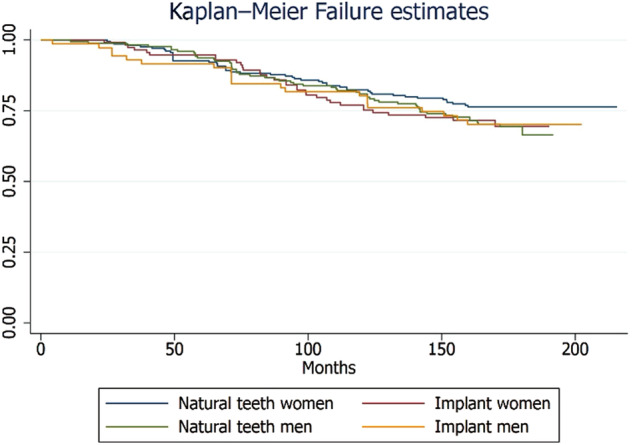
Kaplan-Meier Failure (Group) rates according to the crown-based estimates.

**Fig. 2:**
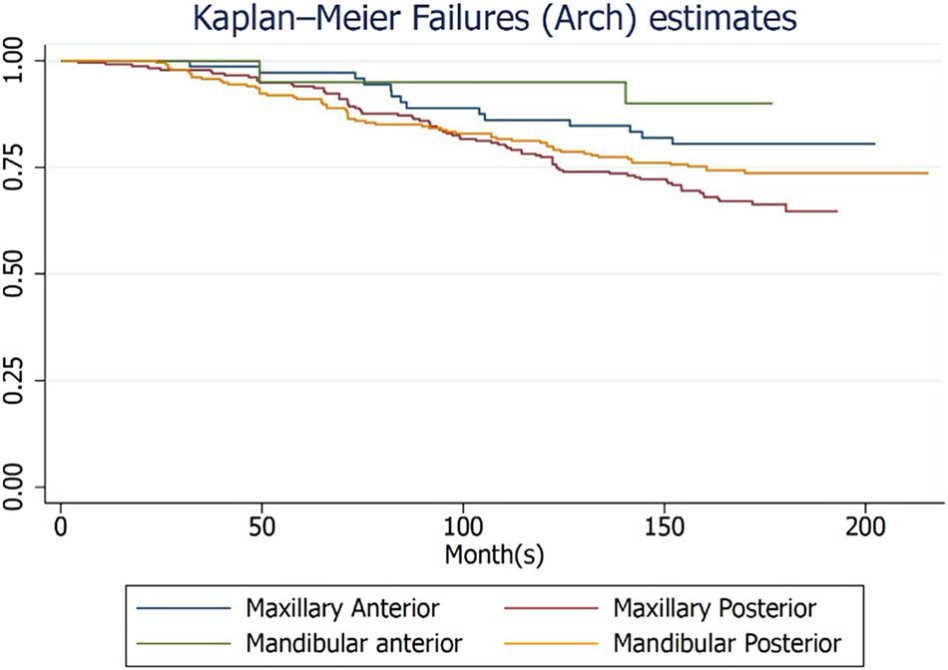
Kaplan-Meier Failure (Arch) rates according to the crown-based estimates.

**Fig. 3:**
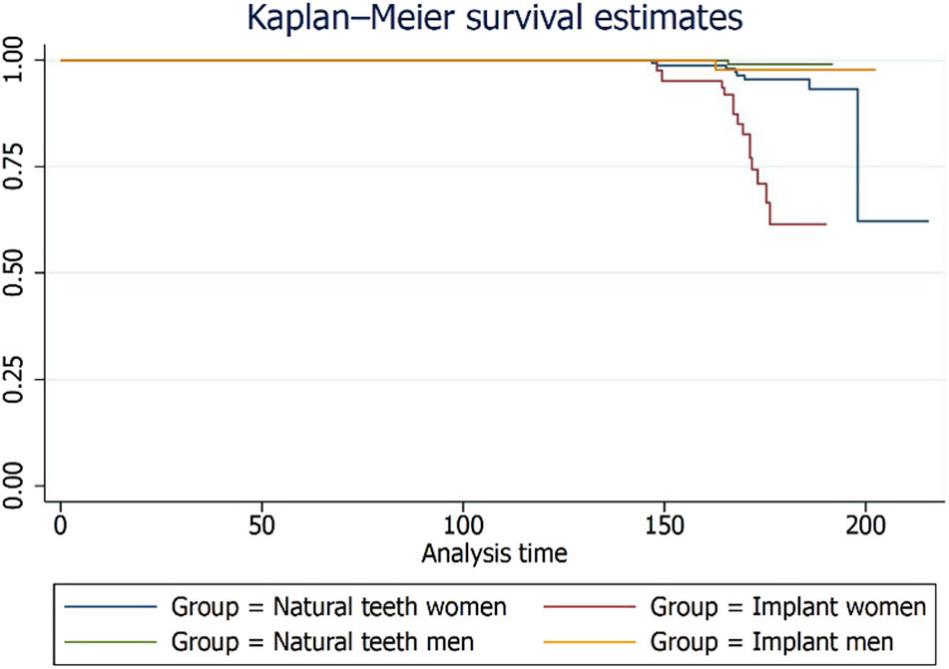
Kaplan-Meier Complications (Group) rates according to the crown-based estimates.

**Fig. 4:**
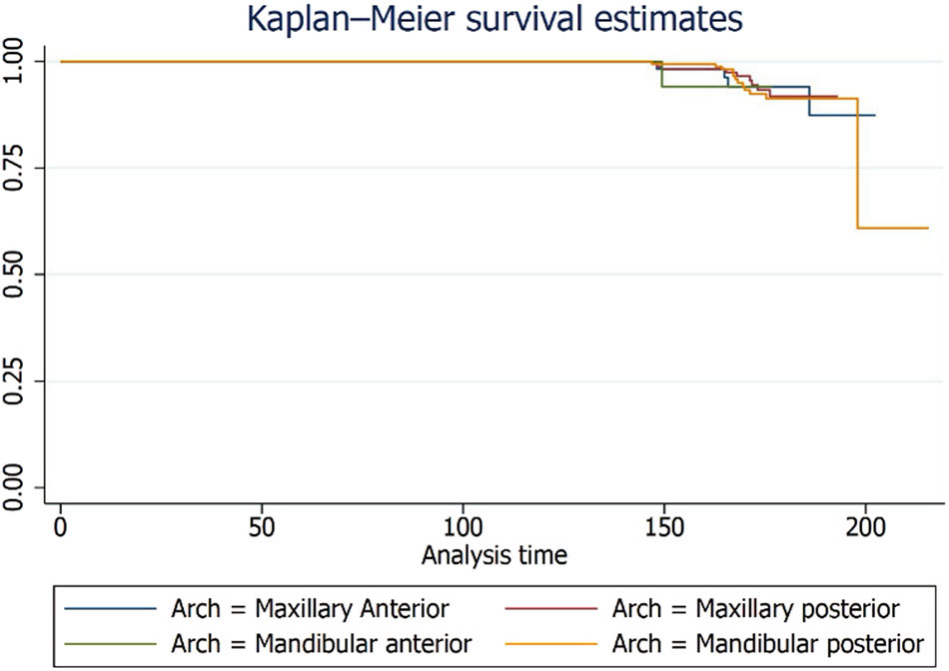
Kaplan-Meier Failure (Arch) rates according to the crown-based estimates.

The study found that the maxilla had a cumulative failure rate of 31.04%, while the mandible had a rate of 25.53% for zirconia crowns, with no statistically significant difference (Supplementary Table S1). Figures 1 to 4 illustrate the failure and complication rates for each crown level analysis. The crown level analysis revealed an overall failure rate of 28.33% for zirconia crowns after 15 years (Table 3). Additionally, the study reported a complication rate of 8.47% (Table 4). The findings showed that the highest failure rates occurred in natural teeth of women and were most prevalent in the posterior region of the maxillary arch, followed by the mandibular posterior region (Figs. 1 and 2). Complications were more commonly observed in women with natural teeth and implant-supported restorations, with a higher incidence in the mandibular posterior region.

**Table 3:** Overall Failure rates for the zirconia crowns from crown-level data.

Intervals (Months)		Total subjects	Events	Loss to follow-up	Cumulative Failure	Crowns without complications	SE	[95% conf. int.]
0	20	562	3	0	0.0053	0.9947	0.0031	0.9835, 0.9983
20	40	558	16	0	0.0339	0.9661	0.0076	0.9474, 0.9783
40	60	542	19	0	0.0677	0.9323	0.0106	0.9081, 0.9502
60	80	523	31	0	0.123	0.877	0.0139	0.8469, 0.9015
80	100	492	23	0	0.164	0.836	0.0156	0.8027, 0.8642
100	120	469	17	0	0.1943	0.8057	0.0167	0.7705,0.8361
120	140	452	18	2	0.2265	0.7735	0.0177	0.7366, 0.8060
140	160	432	21	32	0.2655	0.7345	0.0187	0.6957, 0.7692
160	180	379	6	263	0.2833	0.7167	0.0196	0.6761, 0.7531
180	200	110	1	105	0.2958	0.7042	0.0229	0.6567, 0.7465
200	220	4	0	4	0.2958	0.7042	0.0229	0.6567, 0.7465

**Table 4:** Overall Complications occurred from zirconia crown-level data.

Intervals (Months)		Total subjects	Event	Loss to follow-up	Cumulative Complication	Crowns without complications	Error	[95% conf. int.]
0	20	562	0	3	0	1	0	−
20	40	558	0	16	0	1	0	−
40	60	542	0	19	0	1	0	−
60	80	523	0	31	0	1	0	−
80	100	492	0	23	0	1	0	−
100	120	469	0	17	0	1	0	−
120	140	452	0	20	0	1	0	−
140	160	432	6	47	0.0147	0.9853	0.006	0.9676, 0.9934
160	180	379	18	251	0.0847	0.9153	0.0168	0.8755, 0.9428
180	200	110	2	104	0.1162	0.8838	0.0273	0.8174, 0.9271
200	220	4	0	4	0.1162	0.8838	0.0273	0.8174, 0.9271

The crowns were further categorized into single and multiple crowns. Out of the total number of single and multiple zirconia crowns, the failure rate was 22.4% for 192 single crowns, 30.4% for 46 2–3 unit bridges, 37.0% for 27 4–6 unit bridges, and two out of five >6 unit bridges.

The different types of crown-based failure include veener fractured 5.01% (*N* = 28), zirconia that contains led to loss of retention/re-cementation 14.85% (*N* = 86) and loss of crown due to extraction 1.73% (*N* = 10) (Table 5).

**Table 5:** Reasons of Failures.

Type failure	Freq.	Percent
No failure	438	77.99
Loss of retention or recementation	86	15.31
Veener fractured	28	4.98
Loss of crown due to extraction	10	1.72
Total	562	100

**Fig. 5:**
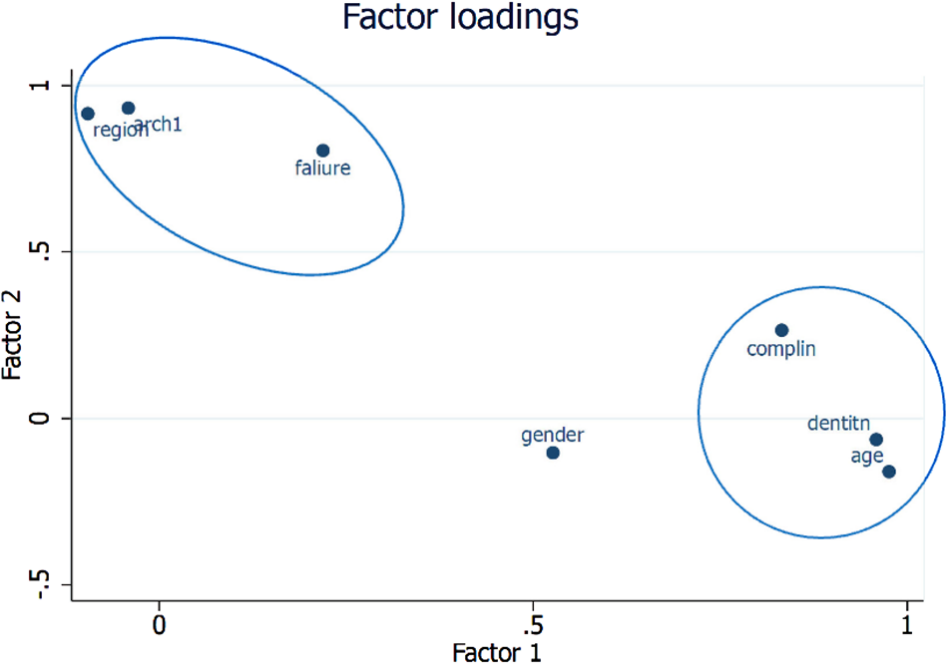
Factor loading plot cluster analysis. Region: Anterior, posterior; Arch: Maxilla and mandible; Complin: Complications; Dentition (Dentitn): Natural teeth and dental implants.

The factor loading plot (cluster analysis) was illustrated in Fig. 5. The age and type of abutment (natural teeth and implant supported) influences the rate of complication, whereas for failures the arch and region in the arch influences the rates of failures.

**Fig. 6:**
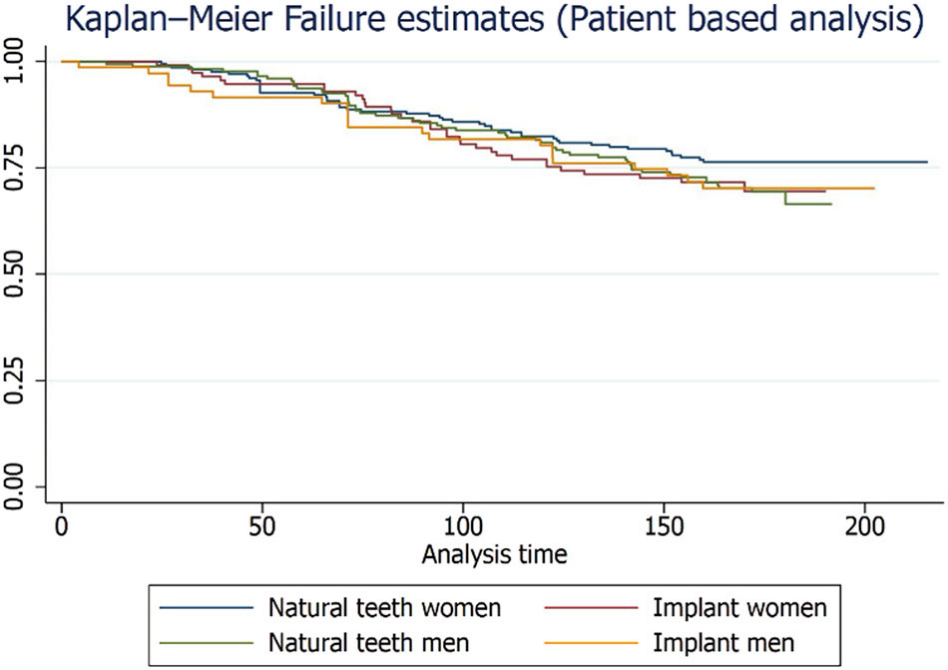
Kaplan-Meier Failure (Group) Patient based analysis.

**Fig. 7:**
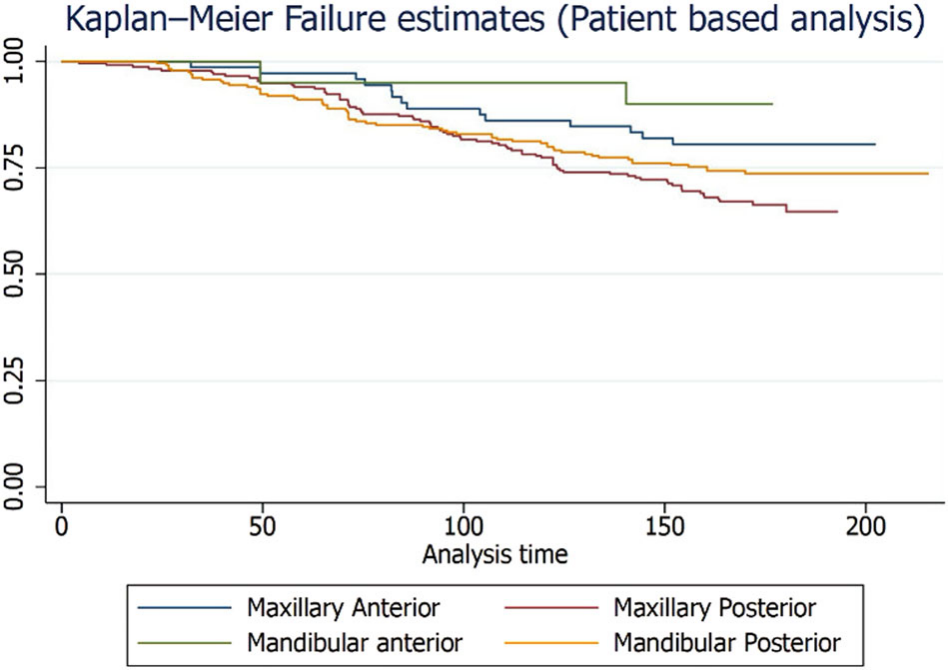
Patient level failure rates among zirconia crowns by Arch analysis.

**Fig. 8:**
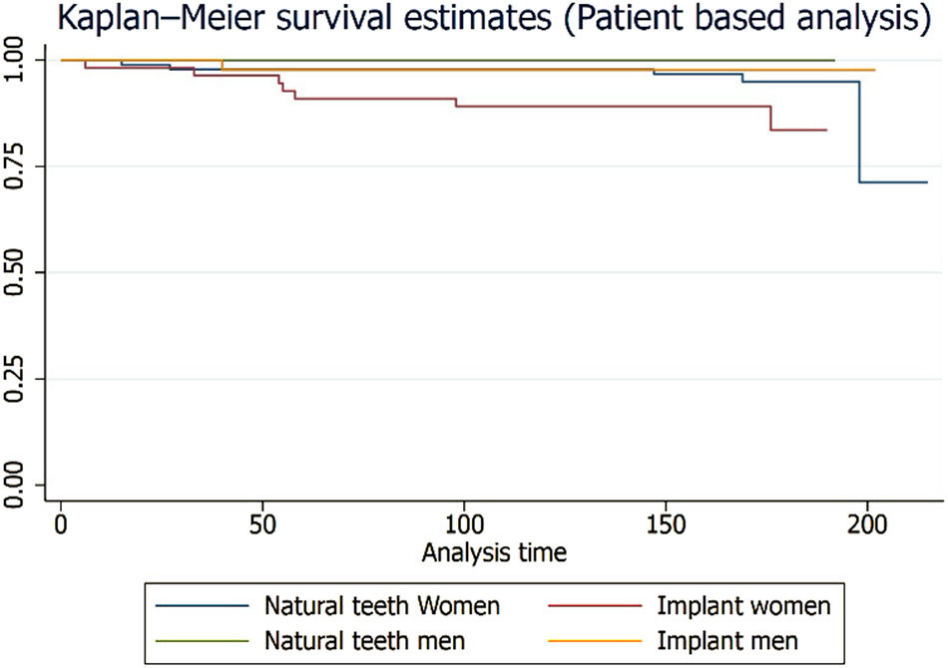
Patient level zirconia crowns complication by Group.

**Fig. 9:**
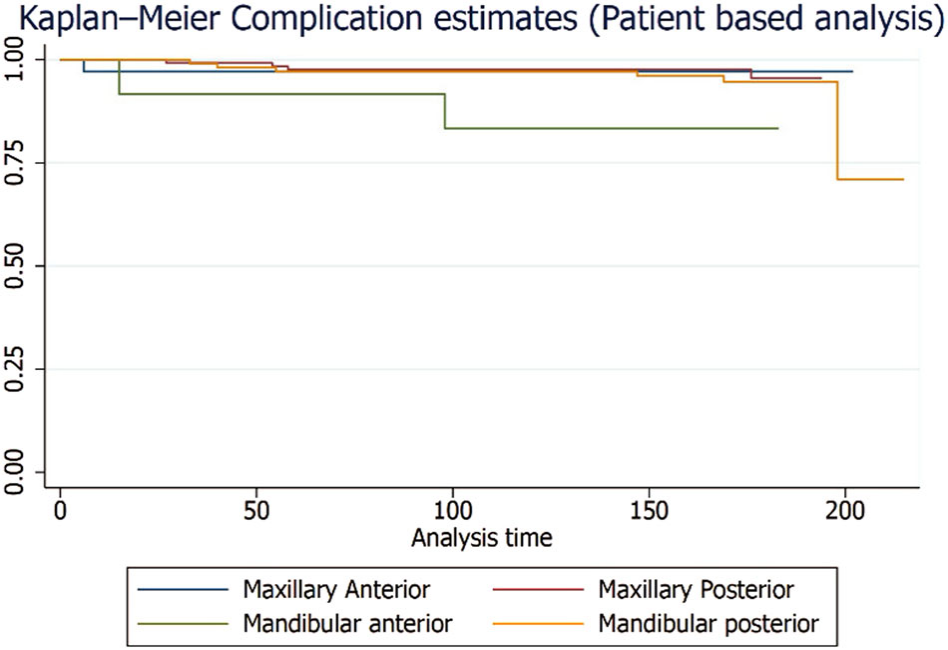
Patient level zirconia crown complication analysis by Arch.

A total of 276 patients were included for patient-level analysis. At the patient level, the failure rate of zirconia crowns was 24.6% and the complication rate was 4.8% over a 15-year period. The distribution and frequency of failures and complications for single and multiple crowns on natural teeth and implants are presented in Supplementary Tables S3 and S4, respectively. The failure and complication rates for single and multiple crowns on natural teeth and implants are shown in Supplementary Table S5. Figures 6 and 7 illustrate the Kaplan-Meier failure rates for zirconia crowns according to group and arch (patient level). Additionally, Figs. 8 and 9 present the analysis of crown complications by group and arch. Descriptive analysis of failures and complications based on individual patient data is provided in the Supplementary Tables file. The life table estimates an overall failure rate of 34.46% and a complication rate of 4.82%.

## Discussion

The therapeutic effectiveness of zirconia-based crowns in clinical trials was investigated by a recent systematic review. According to the findings of 16 clinical studies, implant-supported crowns had a cumulative 5-year survival rate of 97.1% while tooth-supported crowns had a cumulative 5-year survival rate of 95.9%. The most frequent causes of failure for implant-supported crowns were technical (veneering material fractures). Both biologic (endodontic/ periodontitis) and technical (veneering material fractures, loss of retention) reasons for failure were frequent for tooth-supported crowns. Veneering material fractures and bleeding during probing were the most frequent problems with implant-supported crowns [18]. The most frequent issues with tooth-supported crowns included loss of retention, endodontic therapy, material fractures during veneering, and bleeding during probing [18]. The maximum period of follow-up reported in this review was 5-8 years.

Zirconia is a popular material for dental crowns due to its biocompatibility, durability, and esthetics. Several studies have evaluated the survival rates of zirconia-based single crowns and multiple fixed dental prostheses (FDPs) to assess their long-term clinical performance. A systematic review of 35 studies found that zirconia-based single crowns had a high survival rate of 98.3% over an average follow-up period of 5 years [19]. Another systematic review and meta-analysis [20] of 17 studies found that multiple zirconia fixed dental prosthesis (FPD’sP had an acceptable survival rate of 95% over an average follow-up period of 7 years. A retrospective study [21] of 289 study participants with zirconia-based restorations, including 400 FDP’s, found 85.0% favorable long-term clinical performance over a 5–10-year follow-up period [19–21]. However, the success of any dental restoration depends on several factors, including patient factors, operator skill, and maintenance of oral hygiene. Further long-term studies from different groups are needed to confirm the findings and evaluate the performance of zirconia-based restorations in different clinical situations.

In our study, examined the 15-year failure and complication rates of zirconia fixed prosthesis in both tooth-supported and implant-supported cases. To provide a comprehensive analysis, evaluated failure rates at both the patient and crown levels. Our findings indicated that the overall failure rate over the 15-year period at the patient level was 34.4%, with a complication rate of 4.8%. At the crown level, found a failure rate of 28.3% and a complication rate of 11.6%. These results highlight the importance of considering both patient and crown levels in evaluating the success of zirconia fixed prosthesis. Analysing the survival and complication rates of zirconia-based and metal-ceramic implant-supported single crowns was the focus of a review by Pjetursson [19], which had a similar objective to a previous systematic review and a clinical study. The author calculated the failure and complication rates using meta-analysis over a five-year period. According to the systematic review and meta-analysis findings, the 5-year survival rate for single crowns (SCs) supported by zirconia implants (*n* = 912) was 97.6% (95% CI: 94.3–99.0). In contrast, our study found a 15-year survival rate of 67.98% for the maxilla and 74.87% for the mandible (with no significant difference between the two). Similarly, the cumulative survival rate was 72.36% for natural teeth and 69.11% for dental implants. The most common complication was the loss of retention on implants, which could be due to the clinical decision to use temporary cement. Therefore, it is possible to hypothesize that using permanent cement for implant-supported crowns may improve the long-term clinical outcomes. Screw-retained monolithic zirconia implant-supported restorations with CAD/CAM titanium abutments in the posterior region during a 1-year follow-up were studied by Donker et al. and had survival rate of 100% for both implants and restorations with low technical and biological complications [22].

The common complications associated with teeth-supported restorations include loss of retention, veneer fracture, extraction, caries, and other issues. The risk of veneer chipping in such reconstructions is influenced by various clinical parameters, including material-specific factors and forces of mastication. Implants have been shown to have 8.7 times lower tactile sensitivity than natural teeth, requiring greater resistance to masticatory forces. In addition, intraoral circumstances such as temperature and pH variations, as well as material flaws from veneering techniques, can also increase the risk. In our study, found that loss of retention or recementation occurred in 15.31% (*N* = 86) of overall cases, while veneer fracture occurred in 4.98% (*N* = 28), and loss of crown due to extraction occurred in 1.72% (*N* = 10). The most common type of failure experienced by patients over a 15-year period was loss of retention of metal type zirconia in 15.31% (*N* = 86) of total cases (Table 5). Prause et al. [23] study evaluated the survival and success rate of veneered zirconia crowns with an anatomically modified framework design after 10 years in function. The results showed a high survival rate of 92.9%, but a relatively low success rate due to technical complications such as chippings and insufficient marginal gaps. Biological complications occurred less frequently. Patient satisfaction was high, and periodontal conditions were comparable to measured values before crown delivery [23]. There are other studies by Goto et al. [24], Badr et al. [14], Guncu et al. [15], Waldecker et al. [25], Gao et al. [26], Gseibat et al. [27]; Cakan and Özcan [28], Hosseini et al. [29] and Le et al. [30] that were conducted recently, but were of shorter follow-up duration.

The durability of tooth-supported dental prostheses depends in large part on how well dental restorations fit. Poor fit can cause the cement junction to dissolve and impair the restoration, which could cause secondary caries or the restoration to become loose [31]. Inflammation of the gingiva and increased bacterial retention may result from crowns with poor marginal fit on subgingivally positioned margins. Svanborg [31] aimed to look at the accuracy of single- and multi-unit zirconia fixed dental prosthesis supported by natural teeth. The results of the study reported that, the fit and accuracy for the combined marginal gap of single crowns and multi-unit FDPs were 83 µm and 59 µm, respectively. The accuracy was 61 µm, and the internal gap fit was 111 µm. The precision of the zirconia restorations was 53 µm, while the fit for the entire gap was 101 µm [30].

One of the limitations of our study includes, firstly, the patients and treatments were not randomized, which may introduce potential biases. Blinding of the assessments would have enhanced the quality of evidence. To mitigate these effects, an independent clinician who was not involved in the treatment of the patients conducted all assessments. Additionally, the accuracy of fit of zirconia crowns was not recorded, which could have validated the success rates and identified any potential contributing factors for failures or complications. Furthermore, future prospective studies should consider confounding factors such as patients’ oral hygiene habits, smoking history, and medical conditions that could influence the outcomes. The challenges faced during collection of data were recoding the actual time of event. The method of collecting prospective data mentioned, involved documenting failures and complications of zirconia crowns on teeth and dental implants after observing patients in a dental office. Because patients would not have walked immediately when failure or complication have occured or it is unlikely, the patient called the dentist immediately after the event. However, the data was not collected at the time the events occurred. Other studies have also been recorded while the patients walk into the practice. This introduces considerations such as recall bias, missed data points, treatment variances, and contextual factors. These factors can influence data interpretation, clinical evidence, and decision making. The reliance on memory and potential recall bias may compromise data interpretation, while the lack of immediate data collection limits the strength of clinical evidence. Furthermore, the delayed data collection may impact decision making by limiting the ability to make informed decisions based on real-time observations and comprehensive understanding of events. Awareness of these limitations is crucial to ensure a thorough analysis of failures and complications in dental practice. To overcome the above limitations, several strategies can be employed. Implementing real-time data collection systems, standardized protocols, and longitudinal follow-up can help minimize recall bias, improve data accuracy, and capture comprehensive information over time. Collaborating with multiple dental offices or institutions and involving a multidisciplinary team can enhance data interpretation, analysis, and decision making. By implementing these strategies, the reliability and validity of the data can be strengthened, leading to more robust clinical evidence and informed decision making in dental practice.

## Conclusion

In conclusion, in 15 years follow up prospective study, zirconia crowns proved to have longer duration of survival and less complication rates. Future clinical studies should be reported with randomization and blinding that would contribute to the evidence.

### Clinical implication

This clinical evidence presents failure rates observed over a 15-year period, providing valuable information for longer-term follow-up. Various factors influence the success of zirconia crowns placed on natural teeth or implant-supported prostheses, for example: the patients’ systemic health, oral hygiene habits, diet, pH levels, and forces of mastication. Our study demonstrated the longevity and positive outcomes of zirconia crowns, which significantly contribute to improving patients’ esthetics and masticatory function. Additionally, this research identifies gaps in knowledge and suggests methods to address these gaps in future protocols for clinical researchers in the field of dentistry.

### Supplementary information

Supplementary Information

## Data Availability

The data is available with the corresponding author and available upon request.
