# Supplementary material for: Fifteen-year recall period on zirconia-based single crowns and fixed dental prostheses. A prospective observational study
Source: BDJ Open. 2024 Jun 20;10:54. doi: 10.1038/s41405-024-00214-7 (PMC11190277; doi:10.1038/s41405-024-00214-7)
Supplement: Supplementary file 1 — Supplementary Information [file 41405_2024_214_MOESM1_ESM.pdf]

Supplementary tables for Crown Level analysis

Table S1: Life table analysis for the region (Failure)

Interval	Crowns	Events	Loss to follow-up	Cummulative failure	Survival	SE	[95% conf. int.]
**Maxilla**							
0 20	306	4	0	0.0131	0.9869	0.0065	0.9655 0.9951
20 40	302	5	0	0.0294	0.9706	0.0097	0.9442 0.9846
40 60	297	8	0	0.0556	0.9444	0.0131	0.9122 0.9651
60 80	289	17	0	0.1111	0.8889	0.018	0.8480 0.9193
80 100	272	18	0	0.1699	0.8301	0.0215	0.7831 0.8677
100 120	254	12	0	0.2092	0.7908	0.0232	0.7408 0.8323
120 140	242	10	2	0.242	0.758	0.0245	0.7060 0.8022
140 160	230	15	23	0.294	0.706	0.0262	0.6510 0.7540
160 180	192	3	126	0.3104	0.6896	0.0273	0.6326 0.7396
**Mandible**							
0 20	256	0	1	0	1	0	. .
20 40	255	11	0	0.0431	0.9569	0.0127	0.9235 0.9759
40 60	244	11	0	0.0863	0.9137	0.0176	0.8719 0.9423
60 80	233	14	0	0.1412	0.8588	0.0218	0.8097 0.8961
80 100	219	5	0	0.1608	0.8392	0.023	0.7881 0.8790
100 120	214	5	0	0.1804	0.8196	0.0241	0.7667 0.8616
120 140	209	8	0	0.2118	0.7882	0.0256	0.7328 0.8335
140 160	201	6	9	0.2358	0.7642	0.0266	0.7070 0.8117
160 180	186	3	137	0.2553	0.7447	0.0282	0.6843 0.7952

Table S2: Life table analysis by the arch (Failure)

Interval	Crowns	Events	Loss to follow-up	Cummulative Failure	Survival	SE	[95% conf. int.]
**Anterior**							
20 40	92	1	0	0.0109	0.9891	0.0108	0.9253 0.9985
40 60	91	2	0	0.0326	0.9674	0.0185	0.9023 0.9894
60 80	89	2	0	0.0543	0.9457	0.0236	0.8744 0.9770
80 100	87	4	0	0.0978	0.9022	0.031	0.8204 0.9479
100 120	83	2	0	0.1196	0.8804	0.0338	0.7945 0.9319
120 140	81	1	0	0.1304	0.8696	0.0351	0.7818 0.9237
140 160	80	4	9	0.1765	0.8235	0.0401	0.7280 0.8880
160 180	67	0	45	0.1765	0.8235	0.0401	0.7280 0.8880
**Posterior**							
0 20	470	4	1	0.0085	0.9915	0.0042	0.9775 0.9968
20 40	465	15	0	0.0405	0.9595	0.0091	0.9372 0.9740
40 60	450	17	0	0.0768	0.9232	0.0123	0.8952 0.9440
60 80	433	29	0	0.1386	0.8614	0.016	0.8267 0.8896
80 100	404	19	0	0.1791	0.8209	0.0177	0.7831 0.8527
100 120	385	15	0	0.2111	0.7889	0.0188	0.7491 0.8232
120 140	370	17	2	0.2474	0.7526	0.0199	0.7109 0.7891
140 160	351	17	23	0.2851	0.7149	0.0209	0.6715 0.7536
160 180	311	6	218	0.3063	0.6937	0.022	0.6482 0.7345

Patient based analysis

Table S3: Distribution of single and multiple crows of N=276 patients on teeth and implants by region and loss during 15 years follow-up

	Single-unit crowns		Multiple-unit crowns					
	Teeth<br>N (loss)	Implants<br>N (loss)	2-3 Units		4-5 Units		6 Units	
			Teeth<br>N (loss)	Implants<br>N (loss)	Teeth<br>N (loss)	Implants<br>N (loss)	Teeth<br>N (loss)	Implants<br>N (loss)
**Anterior**	18 (7)	12 (0)	4 (2)	1 (0)	6 (2)	3 (2)	0 (0)	0 (0)
**Posterior**	132 (39)	39 (13)	9 (3)	27 (11)	6 (3)	14 (6)	3 (1)	2 (1)
**Total**	150 (46)	51 (13)	13 (5)	28 (11)	12 (5)	17 (8)	3 (1)	2 (1)

Complications

Table S4: Distribution of single and multiple crows of N=276 patients on teeth and implants by region and complication during 15 years follow-up

	Single-unit crowns		Multiple-unit crowns					
	Teeth<br>N(com)	Implants<br>N(com)	2-3 Units		4-5 Units		6 Units	
			Teeth<br>N(com)	Implants<br>N(com)	Teeth<br>N(com)	Implants<br>N(com)	Teeth<br>N(com)	Implants<br>N(com)
**Anterior**	19 (0)	7 (0)	5 (1)	7 (1)	4 (0)	5 ()	0 (0)	0 (0)
**Posterior**	129 (3)	46 (3)	9 (1)	20 (2)	9 (0)	11 (1)	3 (0)	2 (0)
**Total**	148 (3)	53 (3)	14 (2)	27 (3)	13 (0)	16 (1)	3 (0)	2 (0)

Table S5: Zirconia crown patient based descriptive analysis for both group and arch in failure outcomes

Group	Arch	Number of Crowns	Exit time final (Months)		Time at risk	Failures
		Total (N)	Mean (min-max)	Median	Total Months	N
Natural teeth Women	Maxillary Anterior	29	166.2 (49.4-188.5)	184.6	4820.2	5
	Maxillary Posterior	80	151.1(24.8-193.1)	168.3	12093.7	25
	Mandibular Anterior	8	152.45(49.4-176.9)	167.6	1219.6	1
	Mandibular posterior	87	155.9(26-215.7)	174.7	13571	17
Implant Women	Maxillary Anterior	12	136.1(75.4-175)	162.6	1634.3	4
	Maxillary Posterior	37	140.2(40.7-184.6)	140.2	5188.5	13
	Mandibular Anterior	10	165.8(149.4-176.6)	165.6	1658.5	0
	Mandibular posterior	54	145.78(23.7-190.3)	165.55	7872.4	16
Natural Teeth Men	Maxillary Anterior	25	157.4(32.1-184.2)	167.4	3936.1	5
	Maxillary Posterior	82	146.5(11.1-191.8)	169.2	12018.8	30
	Mandibular Anterior	2	140.4(140.4)	140.4	140.4	1
	Mandibular posterior	65	154.1(40.2-191)	172.1	10022.3	17
Implant men	Maxillary Anterior	6	188.45(168.9-202.4)	187.2	1130.7	0
	Maxillary Posterior	35	149.8(4.3-186)	169.2	5243.1	10
	Mandibular Anterior	2	172.8	172.8	172.8	0
	Mandibular posterior	28	132.55(26.6-189.6)	162.7	3711.4	11

Table S6: Failure analysis patient-based zirconia crowns

Intervals (months)		Total subjects	Events	Loss to follow-up	Patients without failures	SE	Cum.Failure	Hazard	[95% conf. int.]	
0	20	276	3	0	0.9891	0.0062	0.0109	0.0005	0.9667	0.9965
20	40	273	7	0	0.9638	0.0112	0.0362	0.0013	0.9337	0.9803
40	60	266	6	0	0.942	0.0141	0.058	0.0011	0.9071	0.9641
60	80	260	7	1	0.9166	0.0166	0.0834	0.0014	0.8772	0.9438
80	100	252	14	0	0.8657	0.0205	0.1343	0.0029	0.8195	0.9008
100	120	238	3	0	0.8548	0.0212	0.1452	0.0006	0.8074	0.8913
120	140	235	13	1	0.8074	0.0238	0.1926	0.0029	0.7556	0.8493
140	160	221	14	15	0.7545	0.0261	0.2455	0.0034	0.6988	0.8013
160	180	192	17	125	0.6554	0.0319	0.3446	0.007	0.5890	0.7138

Table S7: Zirconia crown patient based descriptive analysis for both group and arch in complication outcomes

Group	Arch	Number of Crowns	Exit time final (Months)		Time at risk	Complications
		Total (N)	Mean (min-max)	Median	Total Months	N
Natural teeth Women	Maxillary Anterior	10	161.7 (49-188)	173.5	1617	0
	Maxillary Posterior	43	169.3(27-194)	172	7283	1
	Mandibular Anterior	5	135.6 (15-176)	167	678	1
	Mandibular posterior	35	175.6 (147-215)	171	6149	3
Implant Women	Maxillary Anterior	7	147.28 (6-183)	165	1031	1
	Maxillary Posterior	19	157.1 (54-184)	168	2986	3
	Mandibular Anterior	5	153.8 (98-176)	166	769	1
	Mandibular posterior	24	162.2 (33-190)	174	3893	2
Natural Teeth Men	Maxillary Anterior	12	174.1 (156-192)	174.5	2090	0
	Maxillary Posterior	41	173.9 (138-191)	175	7132	0
	Mandibular Anterior	1	183	183	183	0
	Mandibular posterior	31	176.4 (158-192)	177	5469	0
Implant men	Maxillary Anterior	6	187.8 (168-202)	186.5	1127	0
	Maxillary Posterior	23	172.3 (156-186)	174	3965	0
	Mandibular Anterior	1	172	172	172	0
	Mandibular posterior	13	159.7 (40-189)	169	2077	1

Table S8: Life table for complication rates at patient-based analysis

Interval		Total Subjects	Event	Loss to follow-up	Patients without complications	se	Cummulative complication rate	Hazard	[95% conf. int.]
0	20	276	2	0	0.9928	0.0051	0.0072	0.0004	0.9713, 0.9982
20	40	274	2	0	0.9855	0.0072	0.0145	0.0004	0.9618, 0.9945
40	60	272	4	1	0.971	0.0101	0.029	0.0007	0.9428, 0.9854
80	100	267	1	0	0.9674	0.0107	0.0326	0.0002	0.9382, 0.9829
120	140	266	0	1	0.9674	0.0107	0.0326	0	0.9382, 0.9829
140	160	265	1	16	0.9636	0.0113	0.0364	0.0002	0.9334, 0.9802
160	180	248	2	170	0.9518	0.0139	0.0482	0.0006	0.9155, 0.9727
